# Establishment of a Mouse Model of *Mycoplasma pneumoniae*-Induced Plastic Bronchitis

**DOI:** 10.3390/microorganisms12061132

**Published:** 2024-06-01

**Authors:** Peng Jin, Lin-Sheng Zhao, Tong-Qiang Zhang, Han Di, Wei Guo

**Affiliations:** 1 Department of Respiratory Medicine, Tianjin University Children’s Hospital (Tianjin Children’s Hospital), Tianjin 300134, China; jinpeng1357@126.com (P.J.);; 2 Clinical School of Pediatrics, Tianjin Medical University, Tianjin 300070, China

**Keywords:** *Mycoplasma pneumoniae*, plastic bronchitis, mouse model, lung disease, lymphatic vessel

## Abstract

Plastic bronchitis (PB) constitutes a life-threatening pulmonary disorder, predominantly attributed to *Mycoplasma pneumoniae* (MP) infection. The pathogenic mechanisms involved remain largely unexplored, leading to the absence of reliable approaches for early diagnosis and clear treatment. Thus, the present investigation aimed to develop an MP-induced mouse model of PB, thereby enhancing our understanding of this complex condition. In the first stage, healthy BALB/c mice were utilized to investigate the optimal methods for establishing PB. This involved the application of nebulization (15–20 min) and intratracheal administration (6–50 μL) with 2-chloroethyl ethyl sulfide (CEES) concentrations ranging from 4.5% to 7.5%. Subsequently, the MP model was induced by administering an MP solution (2 mL/kg/day, 10^8^ CFU/50 μL) via the intranasal route for a duration of five consecutive days. Ultimately, suitable techniques were employed to induce plastic bronchitis in the MP model. Pathological changes in lung tissue were analyzed, and immunohistochemistry was employed to ascertain the expression levels of vascular endothelial growth factor receptor 3 (VEGFR-3) and the PI3K/AKT/mTOR signaling pathway. The administration of 4.5% CEES via a 6 µL trachea was the optimal approach to establishing a PB model. This method primarily induced neutrophilic inflammation and fibrinous exudate. The MP-infected group manifested symptoms indicative of respiratory infection, including erect hair, oral and nasal secretions, and a decrease in body weight. Furthermore, the pathological score of the MP+CEES group surpassed that of the groups treated with MP or CEES independently. Notably, the MP+CEES group demonstrated significant activation of the VEGFR-3 and PI3K/AKT/mTOR signaling pathways, implying a substantial involvement of lymphatic vessel impairment in this pathology. This study successfully established a mouse model of PB induced by MP using a two-step method. Lymphatic vessel impairment is a pivotal element in the pathogenetic mechanisms underlying this disease entity. This accomplishment will aid in further research into treatment methods for patients with PB caused by MP.

## 1. Background

Plastic bronchitis (PB) is hallmarked by the development of bronchi-resembling casts within the bronchial lumen, culminating in obstructed ventilation and severe respiratory discomfort, posing a formidable threat to life [1]. Historically, PB was perceived as a rarity, predominantly linked to the aftermath of Fontan procedures in congenital heart disease patients [2,3]. Conversely, accumulating evidence now suggests that PB is more prevalent than previously assumed, with a strong correlation with pulmonary infections [4], notably *Mycoplasma pneumoniae* (MP)-induced pneumonia [4,5]. Presently, scholarly endeavors in PB predominantly revolve around diagnostic and therapeutic strategies, while the comprehension of its fundamental mechanisms remains in its infancy. Consequently, the establishment of an animal model to delve into its pathogenesis has become imperative.

In an antecedent investigation [6], the sulfur mustard mimic 2-chloroethyl ethyl sulfide (CEES) was employed to induce a PB model in rats via aerosolized inhalation, yet this approach overlooked MP infection, deviating from clinical realities. Consequently, the present endeavor aimed to refine the model by transitioning from rats to mice and incorporating MP infection, thereby aligning the model more closely with the clinical phenotype.

PB potentially originates from lymphatic effusion secondary to MP infection, implicating a scenario of compromised lymphatic vessel integrity and functional impairment [7,8,9,10,11]. Nonetheless, the precise signaling cascade orchestrating this mechanism remains to be elucidated. Vascular endothelial growth factor receptor 3 (VEGFR-3), a receptor tyrosine kinase encoded by the *FLT4* gene, plays a significant role in the morphogenesis and maintenance of lymphatic vessels [12]. The VEGFR-3, expressed on lymphatic endothelial surfaces, stimulates lymphangiogenesis via promoting cellular proliferation, migration, and survival mechanisms, which are mediated by the activation of the intracellular phosphatidylinositol 3-kinase (PI3K)/protein kinase B (AKT)/mechanistic target of rapamycin (mTOR) signaling cascade [13,14,15]. Immunohistochemical analysis was utilized to ascertain the expression of VEGFR-3 and delineate the activity of the PI3K/AKT/mTOR signaling cascade within the model, thereby elucidating the intricate association between plastic bronchitis and lymphatic vasculature anomalies.

## 2. Materials and Methods

### 2.1. Animals

Forty-two healthy male BALB/c mice, aged six weeks and weighing 25 ± 1 g, were obtained from Huafukang Biotechnology Co., Ltd. (Beijing, China) and housed at the Experimental Animal Center of the Institute of Radiology, Chinese Academy of Medical Sciences (Tianjin, China). The mice were kept in a specific pathogen-free animal facility maintained at a temperature of (21 ± 3) °C with a relative humidity of 60–70% and a 12-h light–dark cycle. They received standard mouse feed every two days, access to purified water, bedding changes, and cage cleaning, which were performed twice weekly. This study received approval from the Experimental Animal Ethics Committee of Tianjin Children’s Hospital (2023-IITKY-006).

### 2.2. Main Reagents and Instruments

*Mycoplasma pneumoniae* freeze-dried powder (ATCC 15531) was acquired from the United States Bacterial Collection Center. Chemical reagents, such as CEES and anhydrous ethanol, were sourced from Meryer Biochemical Technology Co., Ltd. (Shanghai, China), while tert-amyl alcohol was obtained from Aladdin Biochemical Technology Co., Ltd. (Shanghai, China). The anesthetic tribromoethanol was procured from Shanghai TCI Chemical Industry Development Co., Ltd. (Shanghai, China). A hematoxylin–eosin staining (HE) kit was obtained from Solarbao Technology Co., Ltd. (Beijing, China). The primary antibodies employed comprised rabbit-derived against VEGFR-3 (GB112339), PI3K (GB11769), AKT (GB13011-2), and mTOR (GB111839), alongside a secondary antibody, HRP goat anti-rabbit IgG (GB23303), all sourced from Wuhan Servicebio Technology Co., Ltd. (Wuhan, China). The main instruments, such as the Leica DM400B biological microscope, paraffin embedding machine, and slicing machine, were obtained from Leica Company (Wetzlar, Germany).

## 3. Experimental Methods

### 3.1. Animal Grouping and Model Production

#### 3.1.1. Animal Grouping

**Figure 1:**
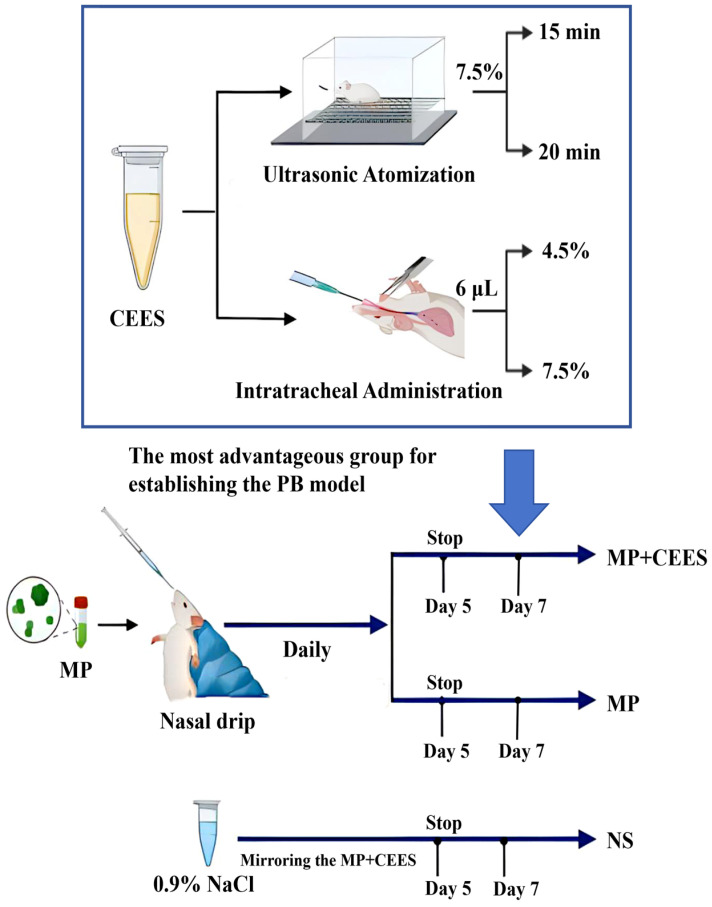
Experimental technology roadmap. The optimal method was selected from four distinct modalities to constitute the pure CEES intervention group for PB model induction. We proceeded to employ this selected modality on the seventh day following the establishment of the MP model, thereby giving rise to the MP+CEES composite model. The control group underwent an identical procedural regimen to that of the MP+CEES cohort, with the substitution of 0.9% physiological saline as the intervention agent.

A total of 42 mice were randomly allocated to seven distinct groups (*n* = 6). In the preliminary stage, the objective was centered on pinpointing the most advantageous group for establishing the PB model, comprising four distinct experimental groups. These groups underwent varied exposures, including continuous nebulization with 7.5% CEES for durations of 15 and 20 min, as well as separate administrations of 6 μL of 4.5% and 7.5% CEES via intratracheal injection. The subsequent stage aimed at establishing two refined groups pertinent to MP infection: an MP-only group, which underwent exclusive intervention with MP, and an MP+CEES group, in which animals were administered CEES after the successful induction of the MP model. The control arm of the study was subjected to the same procedural regimen, receiving only physiological saline (NS) as a comparative measure. Figure 1 visually encapsulates the detailed schema for model establishment and group allocation.

#### 3.1.2. Model Production

(1) The administration method and concentration were as follows:

The 97% CEES solution underwent dilution with anhydrous ethanol in a fume hood, yielding 7.5% and 4.5% CEES solutions, respectively. In the atomization group, mice were intraperitoneally anesthetized with tribromoethanol (0.2 mL/10 g). Subsequently, the atomization group of mice underwent continuous atomization with the 7.5% CEES solution for 15 min and 20 min, respectively. In the tracheal administration group, mice were anesthetized with tribromoethanol, followed by administration of 6 μL of 7.5% and 4.5% CEES solutions through tracheal intubation using a micropipette, respectively. Following administration, the mice were monitored in their cages until they fully recovered from the anesthesia, and their survival status was documented. Respiratory quality, wheezing, and the degree of activity inhibition were assessed according to the clinical performance score table proposed by Veress LA (see Supplementary Materials Table S1) [16]. The scores of each item were totaled to obtain a cumulative score (ranging from 0 to 3 points for each item, with higher scores indicating a worse state, up to a maximum of 9 points).

(2) Method for Modeling *Mycoplasma Pneumoniae*:

The freeze-dried *Mycoplasma pneumoniae* was stored at −20 °C until use and then cultivated at a constant temperature of 27 °C for recovery before inoculation. After administering anesthesia, the mouse’s nasal tip was gently swabbed with a cotton swab soaked in saline solution. The mouse was positioned with its head aligned with its body, perpendicular to the ground. Using a micropipette, MP bacterial solution (2 mL/kg/day, 10^8^ CFU/50 μL) was applied to the mouse’s nasal tip. The mouse was allowed to inhale the bacterial solution naturally as it breathed, directing it into its lungs. The mouse was held mice upright for 15 s, and their breathing, movement, and any nose tip wiping or sneezing were observed immediately after the procedure. This process was repeated daily for five consecutive days. Infected animals were housed separately from uninfected ones.

(3) Production of MP-based Lung PB Model:

Following continuous nasal administration for five days, the MP+CEES group was re-anesthetized with tribromoethanol on the 7th day. Subsequently, 4.5% CEES (6 μL) was instilled into the endotracheal tube using a micropipette. The respiratory status of the mice was monitored after administration.

### 3.2. Lung Fixation

If a mouse shows signs of imminent death, such as a weight loss exceeding 25% or the inability to eat or drink, ethical standards mandate euthanasia before concluding the planned study. Eighteen hours subsequent to their respective CEES dose administration, four CEES cohorts of mice underwent terminal anesthesia, followed by the excision of intact lung samples. In the other groups, comprehensive pulmonary tissue harvests were conducted 18 h after initiation on the seventh day. Mice that died prematurely were immediately dissected upon death. The lung tissue was fixed and preserved in 4% paraformaldehyde tissue fixative.

### 3.3. HE Staining of Lung Tissue

After 24 h of lung tissue fixation, the tissue underwent dehydration, paraffin embedding, and sectioning for pathology. Subsequently, dewaxing and hydration procedures were conducted, followed by staining with HE staining kits. Lastly, the tissue was made transparent and sealed, facilitating the microscopic observation of the lung tissue morphology.

### 3.4. Lung Tissue Pathology Score

Independent pathologists with extensive experience conducted the assessment of lung tissue pathology. The pathology grading system employed a numerical scale ranging from 0 to 26 [17]. Each sagittal section received a score based on the combined evaluation of five categories: (a) the extent of periluminal infiltrate involvement in the bronchiolar and bronchial sites, (b) the severity of periluminal infiltrate, (c) the severity of the luminal exudate, (d) the frequency of perivascular infiltrate, and (e) the severity of parenchymal pneumonia (see Supplementary Materials Table S2).

### 3.5. Immunohistochemistry of Lung Tissue

The specific methods used were as follows: tissue embedding, slicing, dewaxing, and hydration were conducted following the HE staining protocol. Antigen retrieval was performed by heating the tissue in citrate buffer (pH 6.0) in a microwave for 15 min. The tissue was incubated with an adequate volume of endogenous peroxidase blocker at room temperature for 25 min, followed by three consecutive three-minute rinses with PBS buffer (pH 7.4). A sufficient volume of 10% normal goat serum blocking solution was added, followed by overnight incubation at 4 °C with 100 μL of diluted VEGFR-3 (1:500), PI3K (1:1000), AKT (1:200), and mTOR (1:200). Then, 100 μL of enzyme-labeled goat anti-rabbit IgG polymer (1:200) was added and incubated at 37 °C for 20 min. Counterstaining was performed with hematoxylin for 2 min. The transparent slices were sealed. A quantitative evaluation of immunohistochemical staining intensity was conducted employing the ImageJ 1.x software for analysis.

### 3.6. Statistical Analysis

Statistical analysis and chart creation were performed using SPSS 22.0 statistical software and GraphPad Prism 9 software. Measurement data are presented as the mean ± standard deviation. A *t*-test was used to compare two groups, while a one-way analysis of variance (ANOVA) was used for comparisons among multiple groups. A *p*-value of less than 0.05 was considered statistically significant.

## 4. Results

### 4.1. Clinical Manifestations

Following the intervention, in contrast to the control cohort, the CEES group manifested pronounced respiratory insufficiency, whereas the MP group evidenced respiratory infectious manifestations. Upon the completion of a three-day acclimatization phase, the mice demonstrated rapid responsiveness, nimble locomotion, and lustrous pelage. Additionally, they maintained a normal dietary pattern, regular defecation, vibrant claw coloration, and steady respiration.

**Figure 2:**
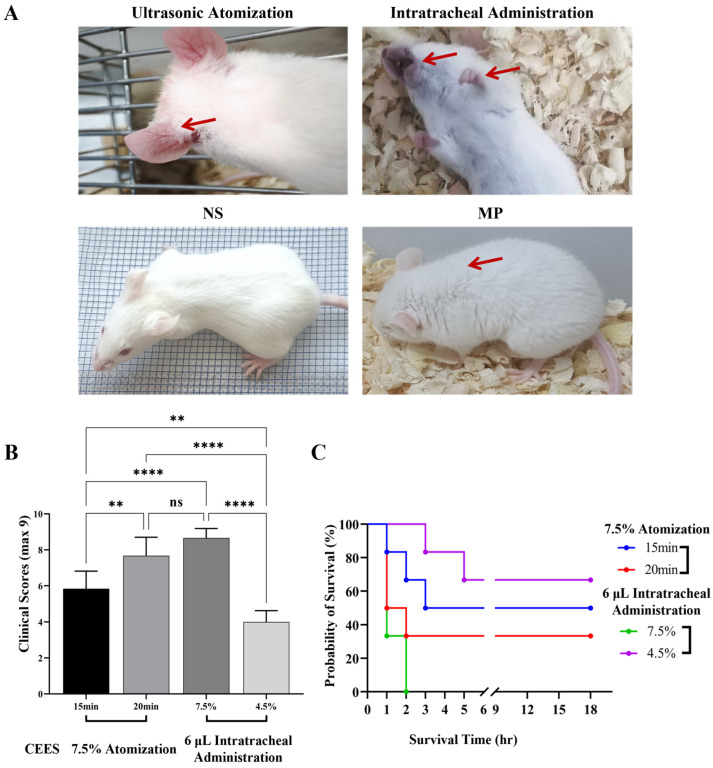
Analysis chart of clinical manifestations, clinical manifestations score, and survival rate of mice. (**A**) In the CEES atomization group, damage to the surface mucosa of mice was evident, including ear hyperemia, as indicated by the red arrow. In the intratracheal administration group, cyanosis of the lips in mice was observed, as shown by the red arrow, indicating hypoxia. The smooth fur of the NS group indicates health, while the upright hair in the MP group, as indicated by the red arrow, suggests the presence of infection. (**B**) Clinical manifestation scores are expressed as the mean ± SD (*n* = 6 mice per group). Statistical significance was denoted as ** for *p* < 0.01, **** for *p* < 0.0001, and ns for non-significant differences (*p* > 0.05). Data analysis was conducted using one-way ANOVA. (**C**) Survival analysis was conducted following the administration of CEES to mice.

After CEES administration, a marked decline in the mice’s health status ensued, typified by apathy toward environmental cues, lethargic behavior, fur dullness, diminished appetite, considerable body mass reduction, intense dyspnea, conspicuous chest heaving, and cyanotic oral, nasal, and claw regions. Mice in the CEES atomization subgroup presented with occluded eyes accompanied by discharge, engorged ear vasculature, and the emission of black stools (depicted in Figure 2A).

The administration of the MP bacterial suspension triggered symptomatic responses in the inoculated mice, including reduced activity levels, piloerection, a decrease in body weight, and repetitive nasal grooming behaviors with claw usage to clean mucoid nasal discharges.

### 4.2. Survival Analysis and Clinical Score Following CEES Administration

The clinical performance scores of the CEES cohorts are depicted as follows (presented in Figure 2B). There were no significant differences between the 20-min atomization group and the 7.5% intratracheal administration group (*p* > 0.05). The clinical manifestations were most severe in the 7.5% CEES intratracheal administration group and least severe in the 4.5% CEES intratracheal administration group. These severity levels were mirrored in the survival rates, with the 7.5% group exhibiting the highest mortality rate, resulting in all mice dying within 2 h after administration. Conversely, the 4.5% group had the lowest mortality rate at 33.3%, and all deaths occurred within 6 h of administration (presented in Figure 2C).

### 4.3. Gross Morphology of Mouse Lungs

**Figure 3:**
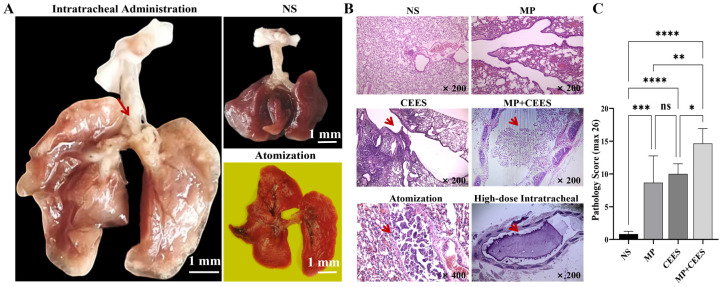
The gross pathological manifestations, H&E staining, and histopathological scores of the lungs in mice with plastic bronchitis. (**A**) Macroscopic pathological observations: In mice administered CEES intratracheally, the trachea presented a white bronchial cast, highlighted by a red arrow. Nebulization group: The whole lungs exhibited a vivid red hue, indicative of profound pulmonary hemorrhage. (**B**) H&E staining findings: In the MP+CEES group, plastic-like material deposition in the airway was evident, as pointed out by a red arrow. CEES nebulization group: Notable features comprised bronchial luminal shedding of epithelial cells, as denoted by red arrows. High-dose intratracheal group: Marked by a red arrow, excessive mucus accumulation in the trachea was provoked. (**C**) Pathological scores are expressed as the mean ± SD (*n* = 6 mice per group). Statistical significance was denoted as * *p* < 0.05, ** *p* < 0.01, *** *p* < 0.001, **** *p* < 0.0001, and ns for non-significant differences (*p* > 0.05). Data analysis was conducted using one-way ANOVA.

Following an 18-h exposure to CEES, our investigation revealed that the mice in the intratracheally administered CEES group exhibited grossly enlarged lungs, displaying a brownish-yellow hue, a hardened and contracted consistency resistant to flattening, and scattered patches of white necrotic areas on the surface. The tracheae in these mice revealed white bronchial casts, accompanied by softened and dilated walls, and either disrupted or absent normal cartilaginous ring architecture. Plastic-like material was observed to line the bronchial passages, obstructing the principal bronchus. It had a soft texture and was difficult to remove (Figure 3A).

Control group mice displayed compact lung parenchyma, characterized by a delicate pink, with tracheae exhibiting transparency and elasticity that allowed for immediate rebound upon stretching to resume their initial configuration. Their airways remained patent, devoid of any plastic or mucus obstructions.

Mice in the nebulized CEES group presented lungs with vibrant red coloration, demonstrating a modest volumetric increase alongside reduced tracheal rigidity. The tracheae in this group were susceptible to injury upon manipulation, and crucially, they lacked evidence of plastic cast formation.

### 4.4. HE Staining

HE staining was performed on mouse lung tissues to visualize and quantify the inflammatory reaction. NS group: The bronchial lumina exhibited a normal morphology, free from inflammatory cellular infiltration or fibrinous exudate, and the alveolar spaces were not infiltrated by inflammatory cells. MP group: Evidence of inflammatory cell infiltration was noted in lung tissue sections. CEES group: Abundant local infiltration of inflammatory cells was observed, leading to inflammatory obstructions within bronchiolar tracts adjacent to the trachea. MP+CEES co-treatment group: Profuse infiltration of red blood cells and inflammatory cells was detected across the visual fields, accompanied by the formation of plastic-like substances in the trachea. Predominantly, the plastic-like substances consisted of aggregations resulting from neutrophilic inflammation coupled with fibrinous exudates. CEES nebulization group: Observations included inflammatory cells, fibrous exudates, and bronchial luminal shedding of epithelial cells; however, PB was not evident. The intratracheal group revealed that excessively high dosages or concentrations elicited copious mucus accumulation in the trachea, obstructing airways, and swiftly inducing asphyxiation and mortality in mice, precluding the development of PB (Figure 3B).

### 4.5. Pathological Score

Mouse lung tissue HE staining was used for pathological scoring and statistical analysis (Figure 3C). Compared with the control group (0.83 ± 0.41), the MP+CEES group (14.67 ± 2.25) showed a significant increase in the lung inflammation score, with a statistically significant difference (*p* < 0.0001). The inflammation score of the 4.5% CEES intratracheal administration group (10 ± 1.55) was higher than that of the control group, with a statistically significant difference (*p* < 0.001). There was no statistically significant difference between the MP group (8.67 ± 4.08) and the CEES group (*p* > 0.05). The pathological inflammation score of the MP+CEES group was higher than that of the MP group and CEES group, and there was a statistical difference (*p* < 0.01).

### 4.6. Immunohistochemistry

**Figure 4:**
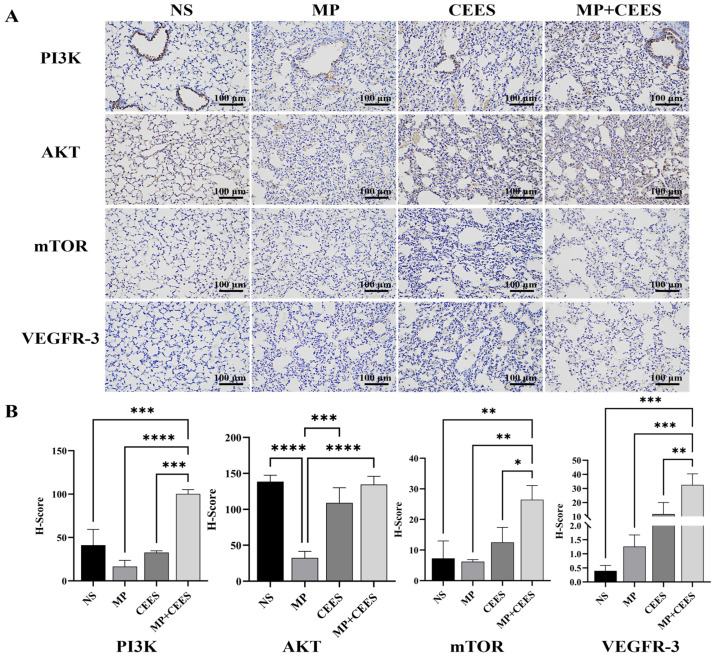
Immunohistochemical results and scoring of PI3K/AKT/mTOR and VEGFR-3 in mouse lung tissue. (**A**) Representative images of immunohistochemical staining in mouse lung tissue. (**B**) Immunohistochemical scores are expressed as the mean ± SD (*n* = 6 mice per group). Statistical significance was denoted as * for *p* < 0.05, ** for *p* < 0.01, *** for *p* < 0.001, **** for *p* < 0.0001. Data analysis was conducted using one-way ANOVA.

This investigation assessed the impact of combined MP+CEES on the PI3K/AKT/mTOR signaling cascade and VEGFR-3 protein expression in murine pulmonary tissue via immunohistochemistry. Specimens stained with DAB and imaged at 200× magnification revealed that, relative to controls, the MP+CEES cohort exhibited a substantial amplification of cytoplasmic PI3K, AKT, and mTOR immunoreactivity, manifesting as intensified dark brown granular staining, suggestive of heightened protein activation states. Image analysis software quantitation disclosed that the PI3K/AKT/mTOR positive area ratio in the experimental group surpassed that of the control by approximately a tripling magnitude, whereas VEGFR-3’s positive area fraction escalated by an eighty-fold increment (Figure 4). These findings suggest that the combination of MP+CEES significantly enhances the activation of pivotal signaling proteins and escalates the expression of lymphangiogenesis-associated proteins, thereby furnishing tangible evidence that fosters a deeper investigation into their modulatory roles within inflammatory responses and lymphatic vessel development processes. 

## 5. Discussion

Presently, the literature implicates a correlation between the onset of PB and a myriad of conditions, including post-operative congenital cardiac disorders, pulmonary inflammation and infections, and anomalies in the lymphatic system of the lungs, among other comorbidities [18]. Independent studies from various single-center institutions consistently identify MP as the primary etiological agent, trailed by adenoviruses and influenza viruses, with bacterial, fungal, and other pathogens being less prevalent [4,19,20]. Despite significant progress in those involving antimicrobial and anti-inflammatory regimens for MP infections, exclusive dependence on these interventions may be insufficient in promptly alleviating the critical health status of pediatric patients or effectively reducing the recurrence of PB episodes [21]. Expanding the understanding of the pathophysiological mechanisms underlying PB is paramount for devising targeted therapeutic interventions. Therefore, there exists a pressing clinical necessity to promptly establish an MP-associated model of PB to facilitate such advancements.

Nevertheless, current animal models of PB are constrained by both a paucity of diversity and inherent limitations. The prevailing literature, as cited in references [6,16,22,23,24], predominantly revolves around sheep models of smoke inhalation burns and rats exposed to CEES-induced models. Regrettably, while the sheep smoke inhalation model does yield PB manifestations, its primary application lies in investigating acute lung injury subsequent to smoke inhalation. The distribution, quantity, and characteristics of PB it induces are atypical. Rat models, while valuable, might not optimally replicate PB secondary to MP infection. Hence, this investigation endeavored to establish a novel PB model explicitly linked to MP infection, addressing the existing clinical and research gap.

BALB/c mice are the preferred animal model for establishing MP infection [25]. Principally, they exhibit heightened susceptibility to MP infection. Furthermore, their status as an inbred strain ensures genetic uniformity, thereby facilitating reproducible experimental outcomes. Additionally, they boast advantageous reproductive traits alongside a prolonged breeding duration. The prevailing protocol for establishing the MP infection model in these mice entails continuous nasopharyngeal instillation of a 50 µL bacterial suspension over a five-day course [26]. Peak pulmonary inflammatory responses are observed within the first week following inoculation [27]. Consequently, BALB/c mice were employed in this study, with subsequent interventions and observations timed to coincide with the apex of pulmonary inflammatory severity, occurring one week after infection. PB peaks at 18 h after CEES exposure in rat models. Thus, this timeframe is optimal for evaluating PB manifestations.

The initial phase of this investigation centered on identifying the most efficacious approach to establish a PB model. Following CEES administration, all groups exhibited a spectrum of respiratory dysfunction, notably featuring tachypnea, heightened ventilation volumes, conspicuous wheezing, and cyanotic episodes. A cited study utilized plethysmography for a more meticulous quantitative evaluation of murine respiratory functional changes [28]. However, its application is constrained by the requirement for the animals to be anesthetized or in a quiescent state [29], limiting broader applicability. After CEES administration, mice in this study exhibited irregular respiration and reduced survival times, rendering them unsuitable for direct respiratory function measurement. Consequently, a less intrusive clinical score for respiratory distress was implemented to quantify impairment, and statistical analyses were conducted to evaluate survival outcomes across groups. The results indicated that in the intratracheal administration group, lowering the dose achieved localized high-concentration stimulation without compromising the entire lung, leading to enhanced survival rates. In the nebulization cohort, a paradoxical outcome emerged: insufficient concentrations failed to elicit a therapeutic effect, whereas excessive concentrations uniformly compromised lung tissue post-administration, escalating mortality rates, thereby illustrating a complex concentration-response paradox.

Following CEES administration, varying extents of diffuse alveolar injury were documented in murine lung tissue across all cohorts, manifesting as alveolar septal edema, thickening, or collapse, augmented erythrocyte presence within alveoli, and infiltrative interstitial pneumonitis. Specifically, the group receiving 6 microliters of 4.5% CEES through intratracheal instillation uniquely manifested bronchial-type airway impediments. Pathological confirmation further elucidated that this phenotype corresponds to Type I plasticity within the SEEAR classification framework [30], characterized chiefly by neutrophil-predominant inflammation accompanied by fibrinous effusion [31]. Within the high-concentration intratracheal group, copious mucus accumulation in the principal bronchi ensued, precipitating asphyxiation and swift fatalities in mice, thereby preempting the development of substantial inflammation necessary for PB manifestation. Mice in the high-concentration nebulized cohorts exhibited vividly discolored, hemorrhagic lungs, coupled with abbreviated survival spans, restricting the potential for inflammatory responses to fully evolve. The low-concentration cohort demonstrated solely histological evidence of airway epithelial shedding, devoid of PB development. Hence, the administration of 6 microliters of 4.5% CEES via the intratracheal route has been established as the paramount intervention strategy.

This study progressed by adopting an MP infection model to elucidate the connection with plastic bronchitis. The virulence of MP is fundamentally attributed to its immunogenic properties [32]. Intranasal administration of inactivated lysed cells to pathogen-free rodents results in interstitial pneumonia and tracheitis that are clinically indistinguishable from those induced by viable mycoplasma [33]. Even in the absence of direct respiratory exposure to MP derivatives, systemic and localized humoral immune responses can still manifest [34]. Relative to controls, the MP+CEES cohort exhibited the maximum histopathological severity score, indicative of exacerbated tissue pathology. This pneumonia is typified by an acute infiltration of neutrophils and lymphocytes enveloping bronchioles, vasculature, and alveolar septa. It is recognized in the literature that *Mycoplasma pneumoniae* pneumonia coincident with plastic bronchitis correlates with modulated expression of inflammatory proteins such as MUC5AC, MUC5B, and layilin [5]. Moreover, MUC5AC and MUC5B are implicated in regulating mucus production in airways, potentially enhancing ciliary clearance dysfunction, thereby positing them as crucial therapeutic targets in deciphering the mechanistic connection between MP infection and plastic bronchitis [35,36].

Elevated inflammatory responses elicited by MP infections are implicated in promoting lymph node enlargement and hyperactivation of the lymphatic system in pediatric populations [37]. Patients diagnosed with *Mycoplasma pneumoniae* pneumonia exhibit a four- to sixfold increased likelihood of experiencing lymph node swelling compared with those afflicted by alternative microbial pneumonia [38]. Dysregulation in lymphangiogenesis coupled with lymphatic vessel dysfunction exacerbates the extent of tissue injury [39,40]. Murine pulmonary infection with MP induces irreversible lymphatic dilation through modulation of the VEGFR-3 signaling axis [41]. The activation of VEGFR-3 signifies a disrupted balance between lymphatic vessel degradation and formation processes [42]. Lymph leakage from impaired lymphatic vessels, compounded by the infiltration of inflammatory cells within the tracheobronchial tree, fosters the progression of type I plastic bronchitis [43,44]. Histological examination of lung tissues from plastic bronchitis patients confirmed the infiltration of lymphatic fluids into the alveolar spaces [45]. This study evaluated the synergistic influence of MP+CEES on the PI3K/AKT/mTOR signaling pathway and VEGFR-3 protein expression in murine lung tissue utilizing immunohistochemical techniques. Compared with controls, the MP+CEES group demonstrated significantly heightened immunoreactivity of PI3K/ AKT/mTOR and VEGFR-3, indicating amplified signaling cascade activity. By contrast, the activation of this signaling cascade was not detected in either the isolated MP group or the CEES group alone. The development of MP-induced plastic bronchitis is potentially ascribed to the selective modulation of lymphatic vessel architectural alterations via the PI3K/AKT/mTOR signaling cascade and the VEGFR-3-mediated pathway.

## 6. Conclusions

This investigation systematically evaluated and identified the optimal methodology for inducing a murine model of plastic bronchitis across varied administration modalities and conditions. Upon co-infection with MP, a model of MP-associated plastic bronchitis was successfully developed. The dual-induced model exhibited characteristic features of profound respiratory impairment, marked weight reduction, and the highest severity of inflammatory injury. Immunohistochemical analyses illuminated that the underlying mechanism of disease induction is potentially attributed to pulmonary lymphatic vessel anomalies, mediated specifically via the PI3K/AKT/mTOR signaling cascade and the VEGFR-3 pathway. These findings facilitate a refined comprehension of the pathogenic mechanisms underlying MP-induced plastic bronchitis. Moreover, the employment of a combined pathogenic factor mediation technique for model induction paves the way for the inclusion of plastic bronchitis cases triggered by diverse etiological agents within the research purview.

Notwithstanding, this study has limitations, including the omission of further sampling mouse blood and bronchoalveolar lavage fluid, the utilization of protein chip assays for MUC5AC and MUC5B quantification, and the specific identification of inflammatory cell subsets via flow cytometric analysis. Future inquiries are warranted to delve further into the intricate mechanisms underlying inflammation’s pathogenesis.

## Data Availability

The raw data supporting the conclusions of this article will be made available by the authors on request.

## Supplementary Materials

The following supporting information can be downloaded at: https://www.mdpi.com/article/10.3390/microorganisms12061132/s1, Table S1: Respiratory Clinical Distress Score Criteria; Table S2: Histopathological Scoring System.

## References

[1] Li Y., Williams R.J., Dombrowski N.D., Watters K., Daly K.P., Irace A.L., Visner G.A., Rahbar R., Fynn-Thompson F. (2020). Current Evaluation and Management of Plastic Bronchitis in the Pediatric Population. Int. J. Pediatr. Otorhinolaryngol..

[2] Healy F., Hanna B.D., Zinman R. (2012). Pulmonary Complications of Congenital Heart Disease. Paediatr. Respir. Rev..

[3] Mackie A.S., Veldtman G.R., Thorup L., Hjortdal V.E., Dori Y. (2022). Plastic Bronchitis and Protein-Losing Enteropathy in the Fontan Patient: Evolving Understanding and Emerging Therapies. Can. J. Cardiol..

[4] Huang F., Gu W., Diwu J., Zhang X., He Y., Zhang Y., Chen Z., Huang L., Wang M., Dong H. (2023). Etiology and Clinical Features of Infection-Associated Plastic Bronchitis in Children. BMC Infect. Dis..

[5] Ma Y., Gu Y., Zhang X., Gu W., Wang T., Sun H., Dai Y., Yan Y., Wang Y., Wang M. (2022). High Expression of MUC5AC, MUC5B, and Layilin Plays an Essential Role in Prediction in the Development of Plastic Bronchitis Caused by MPP. Front. Microbiol..

[6] Veress L.A., O’Neill H.C., Hendry-Hofer T.B., Loader J.E., Rancourt R.C., White C.W. (2010). Airway Obstruction Due to Bronchial Vascular Injury after Sulfur Mustard Analog Inhalation. Am. J. Respir. Crit. Care Med..

[7] Dori Y., Keller M.S., Rome J.J., Gillespie M.J., Glatz A.C., Dodds K., Goldberg D.J., Goldfarb S., Rychik J., Itkin M. (2016). Percutaneous Lymphatic Embolization of Abnormal Pulmonary Lymphatic Flow as Treatment of Plastic Bronchitis in Patients With Congenital Heart Disease. Circulation.

[8] Biko D.M., DeWitt A.G., Pinto E.M., Morrison R.E., Johnstone J.A., Griffis H., O’Byrne M.L., Fogel M.A., Harris M.A., Partington S.L. (2019). MRI Evaluation of Lymphatic Abnormalities in the Neck and Thorax after Fontan Surgery: Relationship with Outcome. Radiology.

[9] Schwager S., Detmar M. (2019). Inflammation and Lymphatic Function. Front. Immunol..

[10] O’Leary C.N., Khaddash T., Nadolski G., Itkin M. (2022). Abnormal Pulmonary Lymphatic Flow on Novel Lymphangiographic Imaging Supports a Common Etiology of Lymphatic Plastic Bronchitis and Nontraumatic Chylothorax. Lymphat. Res. Biol..

[11] Mehta I., Patel K. (2022). Lymphatic Plastic Bronchitis. N. Engl. J. Med..

[12] Hagura A., Asai J., Maruyama K., Takenaka H., Kinoshita S., Katoh N. (2014). The Vegf-C/Vegfr3 Signaling Pathway Contributes to Resolving Chronic Skin Inflammation by Activating Lymphatic Vessel Function. J. Dermatol. Sci..

[13] Deng Y., Zhang X., Simons M. (2015). Molecular controls of lymphatic VEGFR3 signaling. Arterioscler. Thromb. Vasc. Biol..

[14] Wu D., Tian W., Li J., Zhang Q., Wang H., Zhang L., Xie Z., Ji A., Li Y. (2019). Peptide P11 Suppresses the Growth of Human Thyroid Carcinoma by Inhibiting the Pi3k/Akt/Mtor Signaling Pathway. Mol. Biol. Rep..

[15] Kuonqui K., Campbell A.C., Sarker A., Roberts A., Pollack B.L., Park H.J., Shin J., Brown S., Mehrara B.J., Kataru R.P. (2023). Dysregulation of Lymphatic Endothelial VEGFR3 Signaling in Disease. Cells.

[16] Veress L.A., Hendry-Hofer T.B., Loader J.E., Rioux J.S., Garlick R.B., White C.W. (2013). Tissue Plasminogen Activator Prevents Mortality from Sulfur Mustard Analog-Induced Airway Obstruction. Am. J. Respir. Cell Mol. Biol..

[17] Cimolai N., Taylor G.P., Mah D., Morrison B.J. (1992). Definition and Application of a Histopathological Scoring Scheme for an Animal Model of Acute Mycoplasma Pneumoniae Pulmonary Infection. Microbiol. Immunol..

[18] Patel N., Patel M., Inja R., Krvavac A., Lechner A.J. (2021). Plastic Bronchitis in Adult and Pediatric Patients: A Review of Its Presentation, Diagnosis, and Treatment. Mo. Med..

[19] Wang L., Wang W., Sun J.M., Ni S.W., Ding J.L., Zhu Y.L., Ding S.G. (2020). Efficacy of Fiberoptic Bronchoscopy and Bronchoalveolar Lavage in Childhood-Onset, Complicated Plastic Bronchitis. Pediatr. Pulmonol..

[20] Ntiamoah P., Mukhopadhyay S., Ghosh S., Mehta A.C. (2021). Recycling Plastic: Diagnosis and Management of Plastic Bronchitis among Adults. Eur. Respir. Rev..

[21] Zhu Z., Zhang T., Guo W., Ling Y., Tian J., Xu Y. (2021). Clinical Characteristics of Refractory Mycoplasma Pneumoniae Pneumonia in Children Treated with Glucocorticoid Pulse Therapy. BMC Infect. Dis..

[22] Cox R.A., Burke A.S., Soejima K., Murakami K., Katahira J., Traber L.D., Herndon D.N., Schmalstieg F.C., Traber D.L., Hawkins H.K. (2003). Airway Obstruction in Sheep with Burn and Smoke Inhalation Injuries. Am. J. Respir. Cell Mol. Biol..

[23] Houin P.R., Veress L.A., Rancourt R.C., Hendry-Hofer T.B., Loader J.E., Rioux J.S., Garlick R.B., White C.W. (2015). Intratracheal Heparin Improves Plastic Bronchitis Due to Sulfur Mustard Analog. Pediatr. Pulmonol..

[24] Veress L.A., Anderson D.R., Hendry-Hofer T.B., Houin P.R., Rioux J.S., Garlick R.B., Loader J.E., Paradiso D.C., Smith R.W., Rancourt R.C. (2015). Airway Tissue Plasminogen Activator Prevents Acute Mortality Due to Lethal Sulfur Mustard Inhalation. Toxicol. Sci..

[25] Hardy R.D., Jafri H.S., Olsen K., Hatfield J., Iglehart J., Rogers B.B., Patel P., Cassell G., McCracken G.H., Ramilo O. (2002). Mycoplasma Pneumoniae Induces Chronic Respiratory Infection, Airway Hyperreactivity, and Pulmonary Inflammation: A Murine Model of Infection-Associated Chronic Reactive Airway Disease. Infect. Immun..

[26] Wubbel L., Jafri H.S., Olsen K., Shelton S., Barton Rogers B., Gambill G., Patel P., Keyser E., Cassell G., McCracken G.H. (1998). Mycoplasma Pneumoniae Pneumonia in a Mouse Model. J. Infect. Dis..

[27] Hardy R.D., Jafri H.S., Olsen K., Wordemann M., Hatfield J., Rogers B.B., Patel P., Duffy L., Cassell G., McCracken G.H. (2001). Elevated Cytokine and Chemokine Levels and Prolonged Pulmonary Airflow Resistance in a Murine Mycoplasma Pneumoniae Pneumonia Model: A Microbiologic, Histologic, Immunologic, and Respiratory Plethysmographic Profile. Infect. Immun..

[28] Mailhot-Larouche S., Deschênes L., Lortie K., Gazzola M., Marsolais D., Brunet D., Robichaud A., Bossé Y. (2018). Assessment of Respiratory Function in Conscious Mice by Double-Chamber Plethysmography. J. Vis. Exp. JoVE.

[29] Hülsmann S., Khan A., Hagos L., Hindermann M., Nägel T., Dullin C. (2021). Evaluation of a Mechanical Lung Model to Test Small Animal Whole Body Plethysmography. Sci. Rep..

[30] Madsen P., Shah S.A., Rubin B.K. (2005). Plastic Bronchitis: New Insights and a Classification Scheme. Paediatr. Respir. Rev..

[31] Zhang T., Han C., Guo W., Ning J., Cai C., Xu Y. (2021). Case Report: Clinical Analysis of Fulminant Mycoplasma Pneumoniae Pneumonia in Children. Front. Pediatr..

[32] Han C., Zhang T., Zheng J., Jin P., Zhang Q., Guo W., Xu Y. (2022). Analysis of the Risk Factors and Clinical Features of Mycoplasma Pneumoniae Pneumonia with Embolism in Children: A Retrospective Study. Ital. J. Pediatr..

[33] Naot Y., Davidson S., Lindenbaum E.S. (1981). Mitogenicity and Pathogenicity of Mycoplasma Pulmonis in Rats. I. Atypical Interstitial Pneumonia Induced by Mitogenic Myeoplasmal Membranes. J. Infect. Dis..

[34] Jacobs E., Stuhlert A., Drews M., Pumpe K., Schaefer H.E., Kist M., Bredt W. (1988). Host Reactions to Mycoplasma Pneumoniae Infections in Guinea-Pigs Preimmunized Systemically with the Adhesin of This Pathogen. Microb. Pathog..

[35] Hao Y., Kuang Z., Jing J., Miao J., Mei L.Y., Lee R.J., Kim S., Choe S., Krause D.C., Lau G.W. (2014). Mycoplasma Pneumoniae Modulates Stat3-Stat6/Egfr-Foxa2 Signaling to Induce Overexpression of Airway Mucins. Infect. Immun..

[36] Hancock L.A., Hennessy C.E., Solomon G.M., Dobrinskikh E., Estrella A., Hara N., Hill D.B., Kissner W.J., Markovetz M.R., Grove Villalon D.E. (2018). Muc5b Overexpression Causes Mucociliary Dysfunction and Enhances Lung Fibrosis in Mice. Nat. Commun..

[37] Narita M., Yamada S., Nakayama T., Sawada H., Nakajima M., Sageshima S. (2001). Two Cases of Lymphadenopathy with Liver Dysfunction Due to Mycoplasma Pneumoniae Infection with Mycoplasmal Bacteraemia without Pneumonia. J. Infect..

[38] Niitu Y.M. (1983). Pneumoniae Respiratory Diseases: Clinical Features—Children. Yale J. Biol. Med..

[39] Okazaki T., Ni A., Baluk P., Ayeni O.A., Kearley J., Coyle A.J., Humbles A., McDonald D.M. (2009). Capillary Defects and Exaggerated Inflammatory Response in the Airways of Epha2-Deficient Mice. J. Pathol..

[40] Xu X., Greenland J., Baluk P., Adams A., Bose O., McDonald D.M., Caughey G.H. (2013). Cathepsin L Protects Mice from Mycoplasmal Infection and Is Essential for Airway Lymphangiogenesis. Am. J. Respir. Cell Mol. Biol..

[41] Baluk P., Adams A., Phillips K., Feng J., Hong Y.K., Brown M.B., McDonald D.M. (2014). Preferential Lymphatic Growth in Bronchus-Associated Lymphoid Tissue in Sustained Lung Inflammation. Am. J. Pathol..

[42] Zhang L., Ye C., Li P., Li C., Shu W., Zhao Y., Wang X. (2022). Adscs Stimulated by Vegf-C Alleviate Intestinal Inflammation Via Dual Mechanisms of Enhancing Lymphatic Drainage by a Vegf-C/Vegfr-3-Dependent Mechanism and Inhibiting the Nf-Kappab Pathway by the Secretome. Stem. Cell Res. Ther..

[43] Languepin J., Scheinmann P., Mahut B., Le Bourgeois M., Jaubert F., Brunelle F., Sidi D., de Blic J. (1999). Bronchial Casts in Children with Cardiopathies: The Role of Pulmonary Lymphatic Abnormalities. Pediatr. Pulmonol..

[44] Do P., Randhawa I., Chin T., Parsapour K., Nussbaum E. (2012). Successful Management of Plastic Bronchitis in a Child Post Fontan: Case Report and Literature Review. Lung.

[45] Mazza G.A., Gribaudo E., Agnoletti G. (2021). The Pathophysiology and Complications of Fontan Circulation. Acta Biomed..

