# Supplementary material for: Establishment of a Mouse Model of Mycoplasma pneumoniae-Induced Plastic Bronchitis
Source: Microorganisms. 2024 Jun 1;12(6):1132. doi: 10.3390/microorganisms12061132 (PMC11205551; doi:10.3390/microorganisms12061132)
Supplement: Supplementary file 1 [file microorganisms-12-01132-s001.zip › microorganisms-3002807-supplementary.pdf]

Supplementary Materials

Table S1

Table S1. Respiratory Clinical Distress Score Criteria

Score	Respiratory Quality	Stridor	Activity
0	Normal	Normal	Normal
1	Mild abdominal breathing with tachypnea (>100)	Stridor with activity only, mild	Mildly depressed activity,
2	Moderate abdominal breathing. possibly with mild gasping	Stridor at rest, mild to moderate	Moderately depressed activity (movement with stimulation)
3	Severe abdominal breathing. severe gasping, and low respiratory rate(<60)	Stridor at rest, severe	Obtunded, no movement with stimulation; or severe agitation with stimulation

Table S2

Table S2. Histopathological Scoring System

Histopathological scoring system
A. Peribronchiolar/bronchial infiltrates (percentage of sites)
0-none
1-few (<25%)
2-many (25-75%)
3-al1 (>75%)
B. Quality of peribronchiolar/bronchial infiltrates[a)]
0-none
1-mild
2-moderate
3-severe
C. Bronchiolar/bronchial luminal exudate[b)]
0-none
1-minimal
2-heavy
D. Perivascular infiltrate (percentage of sites)
0-none
1-few (<10%)
2-many (10-50%)
3-majority (>50%)
E. Parenchymal pneumonia[c)]
0-none
3-minimal
5-heavy

a) Occasional minimal infiltrates or large peribronchial lymphoid mass seen in normal animals. Mild=abnormal, often with interrupted collar. Moderate= complete collar or crescent collar with <5 cells thickness. Severe=complete collar with >5-10 cells thickness.

b) Minimal=≤25% lumen occlusion. Heavy=>25% lumen occluded.

c) Minimal=patchy parenchymal infiltrates. Heavy=patchy and confluent parenchymal infiltrates.

Numeric score (ranging 0-26) accumulated by the addition of subscores: A+3(average B+average C)+D+E. Average B determined by applying subscore B to all bronchiolar and bronchial airways per sagittal section and determining average. Average C determined by applying subscore C to all bronchiolar and bronchial airways per sagittal section and determining average.
